# Taxon-Driven Functional Shifts Associated with Storm Flow in an Urban Stream Microbial Community

**DOI:** 10.1128/mSphere.00194-18

**Published:** 2018-07-05

**Authors:** Adit Chaudhary, Imrose Kauser, Anirban Ray, Rachel Poretsky

**Affiliations:** a Department of Biological Sciences, University of Illinois at Chicago, Chicago, Illinois, USA; University of Michigan—Ann Arbor

**Keywords:** metagenomics, microbial communities, storm flow, urban streams

## Abstract

Urban streams are susceptible to stormwater and sewage inputs that can impact their ecological health and water quality. Microbial communities in streams play important functional roles, and their composition and metabolic potential can help assess ecological state and water quality. Although these environments are highly heterogenous, little is known about the influence of isolated perturbations, such as those resulting from rain events on urban stream microbiota. Here, we examined the microbial community composition and diversity in an urban stream during dry and wet weather conditions with both 16S rRNA gene sequencing across multiple years and shotgun metagenomics to more deeply analyze a single storm flow event. Metagenomics was used to assess population-level dynamics as well as shifts in the microbial community taxonomic profile and functional potential before and after a substantial rainfall. The results demonstrated general trends present in the stream under storm flow versus base flow conditions and also highlighted the influence of increased effluent flow following rain in shifting the stream microbial community from abundant freshwater taxa to those more associated with urban/anthropogenic settings. Shifts in the taxonomic composition were also linked to changes in functional gene content, particularly for transmembrane transport and organic substance biosynthesis. We also observed an increase in relative abundance of genes encoding degradation of organic pollutants and antibiotic resistance after rain. Overall, this study highlighted some differences in the microbial community of an urban stream under storm flow conditions and showed the impact of a storm flow event on the microbiome from an environmental and public health perspective.

**IMPORTANCE** Urban streams in various parts of the world are facing increased anthropogenic pressure on their water quality, and storm flow events represent one such source of complex physical, chemical, and biological perturbations. Microorganisms are important components of these streams from both ecological and public health perspectives. Analysis of the effect of perturbations on the stream microbial community can help improve current knowledge on the impact such chronic disturbances can have on these water resources. This study examines microbial community dynamics during rain-induced storm flow conditions in an urban stream of the Chicago Area Waterway System. Additionally, using shotgun metagenomics we identified significant shifts in the microbial community composition and functional gene content following a high-rainfall event, with potential environment and public health implications. Previous work in this area has focused on specific genes/organisms or has not assessed immediate storm flow impact.

## Abstract (precis)

Urban streams in various parts of the world are facing increased anthropogenic pressure on their water quality, and storm flow events represent one such source of complex physical, chemical, and biological perturbations. Microorganisms are important components of these streams from both ecological and public health perspectives. Analysis of the effect of perturbations on the stream microbial community can help improve current knowledge on the impact such chronic disturbances can have on these water resources. This study examines microbial community dynamics during rain-induced storm flow conditions in an urban stream of the Chicago Area Waterway System. Additionally, using shotgun metagenomics we identified significant shifts in the microbial community composition and functional gene content following a high-rainfall event, with potential environment and public health implications. Previous work in this area has focused on specific genes/organisms or has not assessed immediate storm flow impact.

## INTRODUCTION

Streams and rivers are important freshwater resources, used for recreation, agriculture, domestic water sources, and industrial purposes. By storing, processing, and transporting terrestrially derived nutrients and organic matter, rivers play an important ecological role in linking biogeochemical cycles between terrestrial and aquatic ecosystems (1). Over the last century, many streams and rivers have witnessed rapid urbanization and anthropogenic development of their drainage basins, which has exposed them to frequent external inputs in the form of wastewater treatment plant (WWTP) effluent, industrial discharge, and sewer/stormwater overflows. These inputs often impact stream hydrological, physicochemical, and biological characteristics (2). For streams and rivers that serve as wastewater and/or stormwater outfall sites, rain-induced storm flow events are especially influential, as they often lead to an increased influx of WWTP effluent and unregulated waste via combined sewer overflows (CSOs) (3, 4). These perturbations bring in nutrients, a variety of microorganisms, including pathogens, and chemical pollutants such as steroid hormones that impact water quality, biodiversity, and ecosystem health (2, 3, 5, 6).

Because urban aquatic streams are typically highly variable systems that are regularly subject to anthropogenic inputs, it is unclear how much isolated perturbations such as rainfall and associated increases in storm flow might influence the water column microbial community, even in the short-term. Studies investigating urban river microbiota using genetic markers for fecal bacteria or 16S rRNA gene-based microbial community surveys have shown the presence of human fecal contamination, “urban signature” bacteria, and changes in community composition in streams and rivers impacted by WWTP effluent, stormwater, and CSOs (7–11). Moreover, others have documented the possible influx of antibiotic-resistant bacteria and pathogens from WWTP effluent (12, 13) and stormwater events (6, 14) into urban environments, further signifying the importance of evaluating the persistence of these organisms and their impact on the riverine microbiome from a public health perspective. While these studies provide valuable information about the effects of storm flow events on urban stream microbial content, they are limited to specific taxonomic and pollutant marker genes. Recent whole-genome shotgun (WGS) metagenomics-based approaches have explored community composition and functional dynamics in urban-impacted streams (15, 16), although a direct effect of storm flow on microbial dynamics remains less explored. A robust evaluation of the impacts of such isolated and short-term perturbations is critical for making predictions about the public health and possible longer-term ecological implications.

In this study, we used both 16S rRNA gene amplicon and shotgun metagenomics to analyze the water column microbial community during base flow and storm flow conditions in the North Shore Channel (NSC) stream, a section of the highly urbanized Chicago Area Waterway System (CAWS) (see Fig. S1 in the supplemental material). We focused on a site downstream of a WWTP and numerous CSO outflow points using 16S rRNA gene amplicon sequencing of samples from both base flow and storm flow over the course of multiple seasons and years. Additionally, samples obtained immediately before and shortly (<24 h) after a single rain event at the same site provided an opportunity for a deep analysis of short-term variability in the taxonomic and functional composition of the water column microbiome using WGS metagenomics. Coupled with the 16S rRNA data from multiple samples, we were able to link some of these changes in the stream microbial taxonomic and functional profiles to storm flow conditions. Although our deep metagenomics-based analysis is centered around a single event, our findings provide a window into the variability and short-term changes in an urban freshwater system and set the groundwork for making predictions about possible ecosystem-level and public-health-related impacts of rainfall events on these systems. Overall, our results show that rain-associated WWTP effluent flow and perhaps CSOs impact the stream microbiome composition and functional potential, with the introduction of exogenous organisms to the system being a significant driver of the observed change.

10.1128/mSphere.00194-18.1 FIG S1  Map of the Chicago Area Waterway System (left panel) and the North Shore Channel (NSC) (right panel). Our study site at NSC is highlighted with an arrow. The point designated WWTP on the right panel represents the O’Brien Water Reclamation Plant. Black dots along the stream represent locations for monitored CSO outfalls. CSO outfalls marked with red stars (locations A, B, and C) recorded CSO events in the evening of 5 October 2013 with durations of 56, 50, and 5 min, respectively (http://www.mwrd.org/irj/portal/anonymous/overview). Download FIG S1, PDF file, 1.9 MB. Copyright © 2018 Chaudhary et al. 2018 Chaudhary et al. This content is distributed under the terms of the Creative Commons Attribution 4.0 International license.

## RESULTS AND DISCUSSION

### Impact of rainfall on NSC microbial community composition.

**FIG 1:**
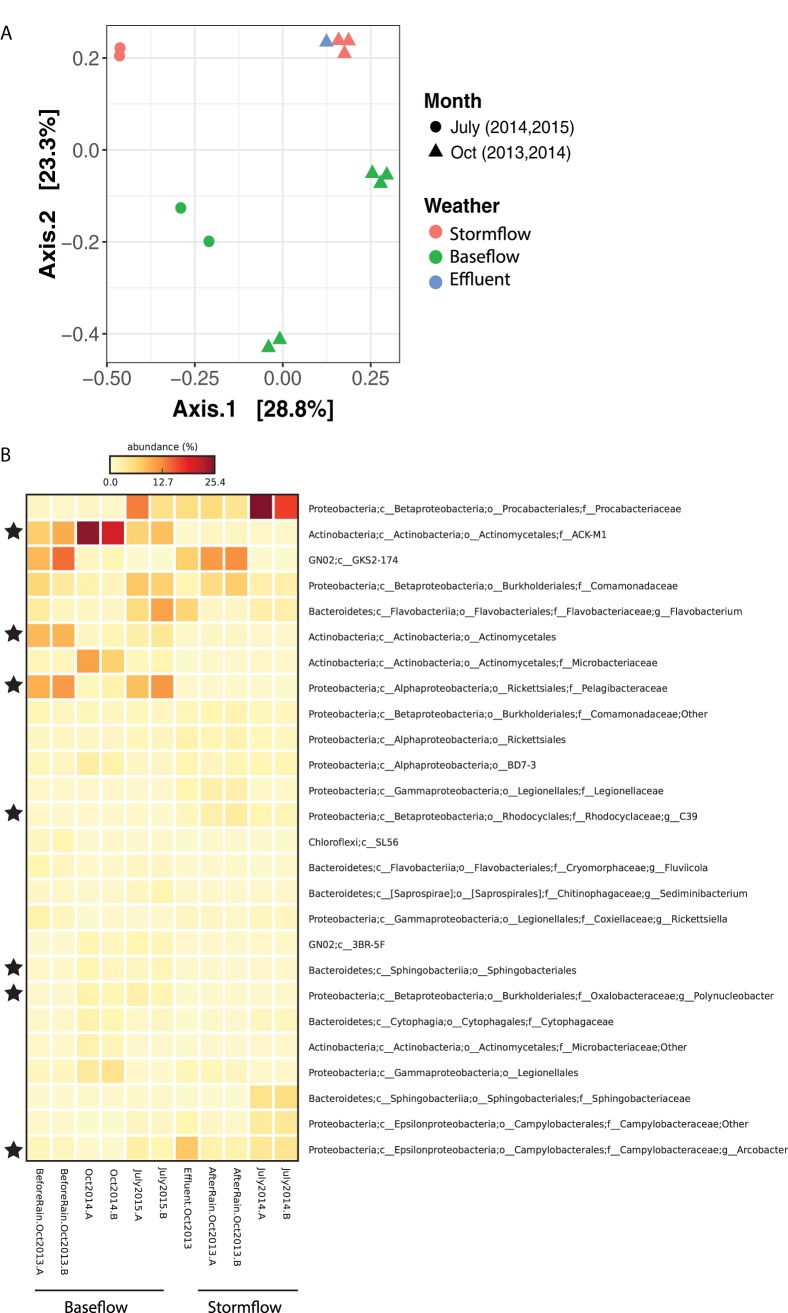
(A) Principal-coordinate analysis (PCoA; Bray-Curtis metric) of OTU-based microbial community diversity for North Shore Channel (NSC) water and WWTP effluent. Samples were obtained during either base flow or storm flow conditions between 2013 and 2015 in the summer (July) and fall (October). Each NSC time point is represented on the PCoA by biological duplicates, except for October 2013 storm flow and base flow samples, which also have sequencing duplicates for one of their biosamples. (B) Heat map representing the relative abundance (percentage of total 16S rRNA gene sequences) of dominant bacterial taxa classified until the lowest possible level (up to genus) for the NSC and effluent samples. Taxa highlighted with a star represent bacterial groups with significantly different relative abundance (*P* < 0.05, Welch’s *t* test) between the storm flow and base flow samples of NSC. Two biological replicates marked as A and B represent each NSC time point, and the average value of these replicates per time point was used in Welch’s *t* test between the two groups (storm flow and base flow).

Rainfall can impact urban waterways by increasing effluent flow from WWTPs or causing combined sewer overflow events (CSOs) at outflow points along streams (4). The NSC site that we investigated has a WWTP (O’Brien Water Reclamation Plant) and several CSO outfall sites within a few kilometers upstream (Fig. S1) and often experiences increased flow from both following rainfall, including the two rain events reported in this study (see Fig. S2 in the supplemental material). Sequences from 16S rRNA gene amplicons at five distinct times between 2013 and 2015 representing both summer and fall and stream base flow (dry weather; three samples) and storm flow (<24 h after rain; two samples) (with additional details in Table S1 in the supplemental material) revealed both a temporal and rainfall-associated clustering of the samples at the operational taxonomic unit (OTU) level (principal-coordinate analysis [PCoA], Bray-Curtis metric) (Fig. 1A). In particular, the separate clustering of storm flow and base flow samples along the principal axis 2 highlights the strong influence of rain on the microbial community composition, regardless of time/year sampled. Such changes might result from either a direct influx of allochthonous microbes or a shift in the resident microbial community in response to altered chemical conditions following rain, although none of the measured physicochemical parameters showed a statistically significant difference between storm flow and base flow conditions (*P* > 0.05, Welch’s *t* test [Table S1]). In addition to shifts in community composition, microbial diversity based on OTU richness and Good’s coverage was slightly higher in the storm flow samples than the base flow samples (see Table S2 in the supplemental material), although the differences were not significant (*P* > 0.05, Welch’s *t* test).

10.1128/mSphere.00194-18.2 FIG S2  O’Brien Water Reclamation Plant effluent flow rate (million gallons per day [MGD]) and rain gauge data for the months of September and October 2013 (http://www.mwrd.org/irj/portal/anonymous/overview). The circled region of the plot corresponds to data around the rain event (5 October 2013), which is the focus of this study. No data were available for 17 September 2013 as the rain gauge was out of service. Download FIG S2, EPS file, 0.8 MB. Copyright © 2018 Chaudhary et al. 2018 Chaudhary et al. This content is distributed under the terms of the Creative Commons Attribution 4.0 International license.

10.1128/mSphere.00194-18.7 TABLE S1  Water chemistry and environmental characteristics for North Shore Channel sampled time points. Download TABLE S1, DOCX file, 0.02 MB. Copyright © 2018 Chaudhary et al. 2018 Chaudhary et al. This content is distributed under the terms of the Creative Commons Attribution 4.0 International license.

10.1128/mSphere.00194-18.8 TABLE S2  Sequencing statistics and diversity estimates for the 16S rRNA gene amplicon libraries used in the study. Download TABLE S2, DOCX file, 0.1 MB. Copyright © 2018 Chaudhary et al. 2018 Chaudhary et al. This content is distributed under the terms of the Creative Commons Attribution 4.0 International license.

To analyze shifts in the microbial community across all storm flow versus base flow samples, OTUs were clustered at various hierarchical taxonomic levels. There was a difference in genus-based community compositions between the storm flow and base flow samples as per analysis of similarity (ANOSIM; Bray-Curtis metric, *R*^2^ = 0.5, *P* = 0.1). Genus-level comparisons of microbial community composition revealed a significantly lower abundance of unknown genera within groups *Pelagibacteraceae*, ACK-M1, and *Actinomycetales* and a significantly higher abundance of *Arcobacter* and genus C39 within the family *Rhodocyclaceae* during storm flow compared to base flow (*P* < 0.05, Welch’s *t* test) (Fig. 1B). The ACK-M1 family of *Actinobacteria* and *Pelagibacteraceae* includes common freshwater organisms that do not favor nutrient-rich conditions (17, 18), while genera within *Rhodocyclaceae* are *Betaproteobacteria*, known to take advantage of nutrient/substrate-rich conditions, likely due to higher growth rates (17). *Rhodocyclaceae* has previously been associated with urban streams and was reported to be abundant in impacted Milwaukee waterways (19). Similarly, *Arcobacter* has often been associated with sewage and WWTP effluent (8, 9, 20). The increase in the relative abundance of these organisms in the NSC following rainfall could be due to point source inputs from the increased effluent flow and/or CSOs and was analyzed in more detail with shotgun metagenomics (described below).

Overall, the rain-associated changes in the microbial community composition appeared to be directly related to increased effluent; the after-rain community OTUs were more similar to those in the WWTP effluent than to those in the before-rain community (Fig. 1A). This could be linked to a few taxa, such as unknown genera within families *Procabacteriaceae* and *Legionellacaea* as well as the genus *Arcobacter*, which were abundant in the effluent and increased in the stream after rain (Fig. 1B).

### Metagenomics-based microbial community composition before and after rain in North Shore Channel.

The overall trends from the 16S rRNA gene-based analysis across seasons and years warranted a whole-community metagenomic analysis of more temporally resolved samples clustered around a large rainfall event. Here, we report our observations of a single, isolated event, acknowledging that this might not be representative of every rainfall event in this dynamic urban system. Instead, our results allow us to make predictions and better understand how urban microbial communities might be influenced by system-wide pertubations. Metagenomes with 4.06 to 16.21 million reads per library were obtained (see Table S3 in the supplemental material) from the same NSC site discussed above (Fig. S1) before and <24 h after a heavy rainfall that followed a dry period in October 2013 (Fig. S2). These were used to comprehensively identify short-term changes in the microbial taxonomic profile after the rain. The rain resulted in increased WWTP effluent flow into the stream for ~24 h following precipitation, from <200 million gal per day (MGD) to >300 MGD, and several CSO events at at least three outfall locations upstream of our sampled site within 10 h of rain (http://www.mwrd.org/irj/portal/anonymous/overview) (Fig. S2). Community coverage estimates using read redundancy (21) showed that the before-rain metagenomes captured between 50 and 60% of the community and the after-rain libraries captured approximately 40% (see Fig. S3 in the supplemental material), indicating only a nominal increase in diversity after rainfall; as described above, a small increase in community OTU richness after rain was also observed with the 16S rRNA gene amplicon data (Table S2). Furthermore, the concentrations of microbial cells in the before- and after-rain samples were determined by DAPI (4′,6-diamidino-2-phenylindole) counts and found to be similar: 1.39 × 10^6^ and 1.25 × 10^6^ cells/ml, respectively. Previous studies have reported conflicting responses of microbial community diversity to urban inputs, with some showing an increase (19) and others a decrease (15, 22) relative to less-impacted conditions/systems. This may be due to different base conditions (operationally defined here as dry weather for at least 72 h); the NSC is characterized by significant urban effluent flow even in the absence of rain. While Lake Michigan provides the primary freshwater input, about 70% of the annual flow through the CAWS is contributed by the treated effluent discharge from WWTPs in the city (23) during both base flow and storm flow conditions. Our results do not show a strong pattern of change in microbial community diversity/richness during storm flow in NSC, perhaps because of the variable nature of urban stream microbial communities or due to the small size of this study. However, we hypothesize based on our results that individual rain events might not significantly impact microbial diversity in this system.

10.1128/mSphere.00194-18.3 FIG S3  Community coverage estimates based on metagenomic reads generated using Nonpareil for the before- and after-rain metagenomes. Sample numbers 1 and 2 for each time point represent biological replicate libraries. Download FIG S3, EPS file, 0.9 MB. Copyright © 2018 Chaudhary et al. 2018 Chaudhary et al. This content is distributed under the terms of the Creative Commons Attribution 4.0 International license.

10.1128/mSphere.00194-18.9 TABLE S3  Sequencing statistics for the metagenomes used in the study. Download TABLE S3, DOCX file, 0.01 MB. Copyright © 2018 Chaudhary et al. 2018 Chaudhary et al. This content is distributed under the terms of the Creative Commons Attribution 4.0 International license.

**FIG 2:**
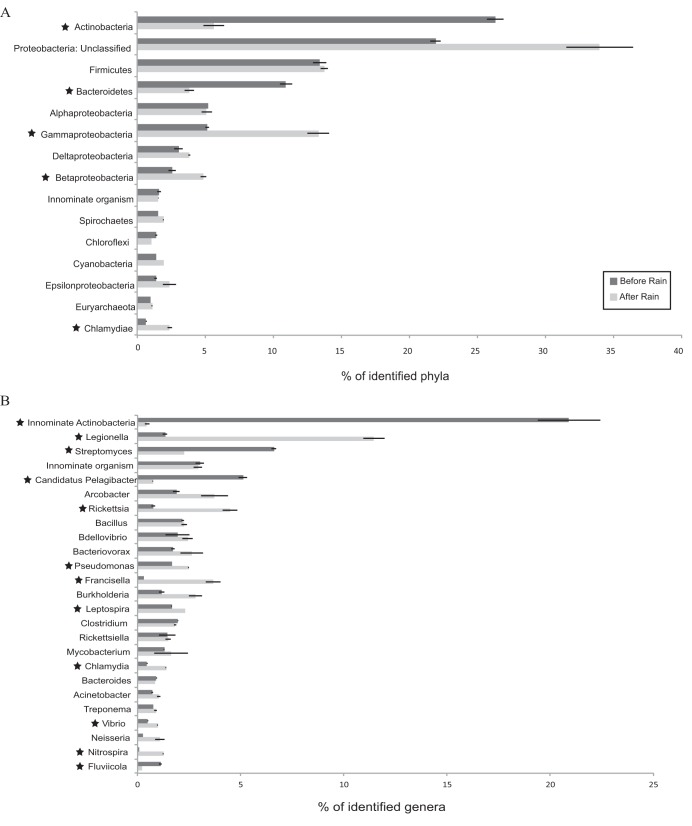
Rank abundance plots for (A) phylum (*Proteobacteria* subdivided into classes)- and (B) genus-level classifications of metagenomic contigs from October 2013 before- and after-rain samples. The relative abundances of different taxa are averages of biological replicates for each sample (*n =* 2). Based on taxon mean relative abundance across the samples, only the top 15 phyla and top 25 genera are shown. Phyla and genera highlighted with a star represent taxa with significant difference in relative abundance between the before- and after-rain microbiota (*P* < 0.05, *t* test, false-discovery rate corrected). “Innominate organism” comprises contigs classified as organisms that either belonged to no known phylum/genus or a candidate phylum/genus.

Despite overall similarities in microbial diversity and cell counts, numerous taxonomic differences were seen following rain, indicating that these changes likely reflect actual changes in microbial populations. The microbial communities pre- and post-rainfall determined both from 16S rRNA genes and by assigning taxa to assembled metagenomic contigs showed overall concordance; however, we focused on the assembled contigs for a high-resolution, population-level characterization of the community and to evaluate possible links between taxonomic and functional changes in the microbiome (24). About ~67% of the large (>500-bp) contigs used by MyTaxa were classifiable at the phylum level, ~35% at the genus level, and 24% at the species level. At the phylum level (*Proteobacteria* divided into subphyla), several individual taxa showed significantly different relative abundances after rain with large effect sizes (Fig. 2A). *Actinobacteria* and *Bacteroidetes* significantly decreased in relative abundance after rain, whereas *Gammaproteobacteria*, *Betaproteobacteria*, and *Chlamydia* significantly increased (*P* < 0.05, *t* test, false-discovery rate corrected) (Fig. 2A). Similarity percentage (SIMPER) analysis (25) revealed that *Actinobacteria*, *Gammaproteobacteria*, and unclassified *Proteobacteria* contributed the most (35, 14, and 21%, respectively) to the differences in community compositions between the before- and after-rain samples at the phylum level. At the genus level, the decrease in relative abundance of innominate (unclassified at genus level) *Actinobacteria*, “*Candidatus* Pelagibacter,” and *Streptomyces* as well as the increase in relative abundance of *Legionella* and *Rickettsia*-affiliated sequences after rain contributed to the major change (>50%) in community composition (Fig. 2B). *Francisella*, *Nitrospira*, *Chlamydia*, and *Pseudomonas* were other major genera that increased significantly (*P* < 0.05, *t* test, FDR corrected) in relative abundance in the after-rain microbiome. As was observed with 16S rRNA amplicons in all samples (described above), the urban signature bacterium *Arcobacter* increased by >50% in relative abundance following rain, although the increase was not statistically significant (Fig. 2B). *Legionella*, *Pseudomonas*, and *Arcobacter* have all been previously associated with effluent contamination of urban waterways (20), supporting the significant role of increased effluent flow on the NSC microbiome. Increases in the relative abundance of other taxa such as *Francisella*, *Rickettsia*, and *Chlamydia* that comprise pathogenic species (26, 27) and are usually not abundant in aquatic environments could be a result of microbial influx from the effluent and/or the CSOs upstream. The decrease in the freshwater groups of *Actinobacteria* and *Pelagibacteria* after rain likely reflects a dilution effect on base flow NSC waters from the increased effluent and CSO flow. Several species, including Francisella tularensis, “*Candidatus* Nitrospira defluvii,” Legionella longbeachae, and Enterococcus faecalis, were rare (<0.1% of the total sequences characterized by MyTaxa) in the before-rain microbiome but increased in relative abundance after rain to >0.1% (Table S3). Most of these species are not common freshwater bacteria and are indicative of contamination.

### Population-level changes in response to rainfall in the North Shore Channel.

We followed population-level trends for abundant organisms that exhibited large changes in their relative abundance after rain. Organisms most similar to Legionella pneumophila increased 10-fold in relative abundance after rain and also comprised the largest fraction of characterized species (11%) in the after-rain microbiome. Reads were recruited to the longest contig assigned to L. pneumophila in the rain-associated samples, with roughly equal similarities (about 90 to 100% nucleotide identity) from each sample, suggesting the presence of the same population both before and after rain that increased substantially after rain (see Fig. S4 in the supplemental material). This was supported by similarities in the average amino acid identity (AAI) of predicted protein-coding genes from L. pneumophila before and after rainfall contigs (60% and 63%, respectively) to the genome sequences of the environmental isolate L. pneumophila strain LPE509 and the clinical isolate L. pneumophila subsp. pneumophila strain Philadelphia 1. The AAI between genes attributed to L. pneumophila in the before- and after-rain metagenomes was 83%. Although genome pairs for the same species typically exhibit higher AAIs (~90%) (28, 29), 83% still signifies close genetic relatedness and not necessarily distinct populations. Overall, these results indicate that the before- and after-rain *Legionella* isolates are members of the same species, but different from any currently known, sequenced members of *Legionella*. The discordance between our *Legionella-*like organisms and well-characterized L. pneumophila strains also makes it unclear if the corresponding populations are pathogenic, although a few predicted genes (1 and 3 for the before- and after-rain metagenomes, respectively) had high identity matches (>90%) to known L. pneumophila virulence genes in the Virulence Factor Database (http://www.mgc.ac.cn/VFs/). Organisms within *Legionella* have been associated with artificial aquatic environments, such as water distribution systems and cooling towers in buildings (30, 31), as well as WWTP effluent (20): thus their dramatic post-rain surge is not surprising.

10.1128/mSphere.00194-18.4 FIG S4  Reads from before-rain (top) and after-rain (bottom) data sets were mapped to the longest contig attributed to Legionella pneumophila from the after-rain metagenome. Reads for biological replicate libraries (*n =* 2) were pooled for both the before- and after-rain time points. Download FIG S4, EPS file, 1.2 MB. Copyright © 2018 Chaudhary et al. 2018 Chaudhary et al. This content is distributed under the terms of the Creative Commons Attribution 4.0 International license.

Another notable increase in relative abundance after rain (~16-fold) was attributed to Francisella tularensis, an organism with known soil- and waterborne pathogenic subspecies (27, 32). Using a similar approach to that described above, AAIs between genes attributed to F. tularensis in before- and after-rain samples and a reference genome of pathogenic subspecies F. tularensis subsp. tularensis SCHU S4 were 47% and 54%, respectively. Similar AAI values were observed between the metagenomic sequences and genomes of low-virulence subspecies of this organism. The AAI between the before- and after-rain F. tularensis genes was 68%. Thus, sequences classified as F. tularensis in our samples likely share the same taxonomic order *Thiotrichales*, but are different species from the known F. tularensis and might represent different populations within the same genus in the before- and after-rain samples, although the low number of sequences in the before-rain data set could bias AAI calculation.

We also evaluated the population dynamics for species that dramatically dropped in relative abundance after the rain. *Actinobacterium* SCGC AAA027-L06 is a member of the ubiquitous freshwater *Actinobacteria* lineage acI-B (33), and the relative abundance of contigs affiliated with this organism decreased dramatically (43-fold) after rain. Read recruitment indicated similarity between the before- and after-rain populations, with reads from each sample sharing ~90 to 100% nucleotide identity to the largest contig of this organism, although fewer reads mapped to the contig from the after-rain samples (see Fig. S5 in the supplemental material). As with the L. pneumophila population, the 84% AAI between the before- and after-rain sequences indicates close genetic relatedness between the two populations. Furthermore, the AAIs with respect to the *Actinobacterium* SCGC AAA027-L06 draft genome were similar for the sequences from the before- and after-rain microbial communities (81% and 83%, respectively), indicating close genetic relatedness to this organism. Members of the acI-B lineage have been detected in diverse freshwater habitats (19, 34–36) and tend to prefer oligotrophic environments due to their small cell size and oligotrophic life strategies (18, 37). Their decrease in relative abundance after rain likely reflects the reduced influence of freshwater flow from Lake Michigan due to increased wastewater flow.

10.1128/mSphere.00194-18.5 FIG S5  Reads from before-rain (top) and after-rain (bottom) data sets were mapped to the longest contig attributed to *Actinobacterium* SCGC AAA027-L06 from the before-rain metagenome. Reads for biological replicate libraries (*n =* 2) were pooled for both the before- and after-rain time points. Download FIG S5, EPS file, 1.3 MB. Copyright © 2018 Chaudhary et al. 2018 Chaudhary et al. This content is distributed under the terms of the Creative Commons Attribution 4.0 International license.

### Overall functional gene content in before- and after-rain microbial communities.

**FIG 3:**
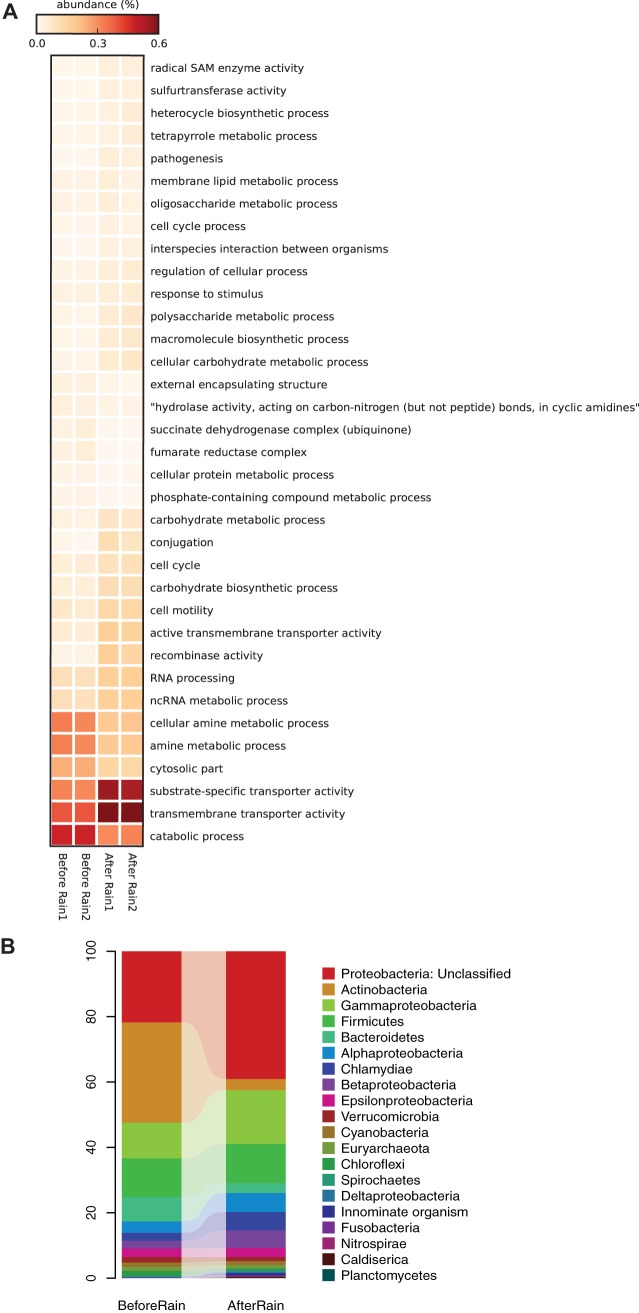
(A) Heat map showing relative abundance (percentage of total predicted genes) at level 3 of Gene Ontology (GO) terms for the before- and after-rain microbiomes. GOs that had a higher relative abundance (>50%) in one of the two groups (before versus after rain) compared to the other are shown. GOs that had less than 100 gene counts (*in situ* abundance) across all the samples have been excluded from the plot. Samples numbered 1 and 2 for each time point represent biological replicates. (B) Taxonomic composition at the phylum level of genes from the rain event microbial communities classified within the GO term “transmembrane transporter activity.” Relative abundances are a fraction of total sequences identified at the phylum level.

Functional gene profiles revealed taxon-driven shifts in the microbial community functional potential after rain. Although many abundant Gene Ontology (GO) terms related to housekeeping functions, such as nucleic acid and small molecule binding, did not significantly change in relative abundance after rain (data not shown), we observed an increase of >50% of functions within the broad terms of transporter activity and carbohydrate metabolism after rain (Fig. 3A). Little is known about the selective increase in transporter genes under various environmental conditions, although transporters are the primary microbial mechanism for the uptake and subsequent assimilation of nutrients and organic matter. Transporter gene expression has been shown to change in response to organic carbon inputs (38) and a phytoplankton bloom (39) in marine systems. In freshwater systems, transporters are important for cyanobacterial phosphorus acquisition (40). More recently, amino acid and amine transporter genes were among those found to be associated with various environmental conditions in *Polynucleobacter* populations in the CAWS (41). Here, we identified transporter genes that were more abundant following the observed rain event and were primarily related to transmembrane and substrate-specific transporter activity (Fig. 3A).

Within the broad GO term of transporter activity, genes related to substrate-specific transmembrane transporter activity, specifically organic acid and ion transmembrane transporter activity, doubled in relative abundance after rain from an average of 0.06% to an average of 0.12% (see Fig. S6 in the supplemental material). Genes encoding all transmembrane transporters were primarily attributed to *Actinobacteria* (31% of the identified sequences at phylum level) and unclassified *Proteobacteria* (22%) before rain, whereas unclassified *Proteobacteria* (39%) and *Gammaproteobacteria* (16%) were the major groups encoding transporters after rain (Fig. 3B). *Gammaproteobacteria* harboring transporter genes increased by 51% after rain, while *Actinobacteria* encoding these genes exhibited more than 9-fold decrease, mirroring the shifts observed for the overall taxonomic profiles for these groups (Fig. 2 and 3B). Genera contributing to the increase in gammaproteobacterial sequences included *Legionella*, *Francisella*, and *Pseudomonas*, exhibiting a pattern similar to the shifts in their relative abundance in the overall microbial community. Furthermore, as with the overall microbial community, *Actinobacterium* SCGC AAA027-L06 (unclassified at genus level) contributed the largest fraction of sequences containing transmembrane transporter activity genes within *Actinobacteria* in the before-rain community. Interestingly, based on the functional gene content of organisms with dominant shifts in their relative abundance, those organisms that increased after rain had a higher proportion of their genes affiliated to transporter functions compared to those that dropped in abundance after rain. For instance, 3.7% and 6.8% of the L. pneumophila and F. tularensis genes, respectively, were associated with transmembrane transport, whereas *Actinobacterium* SCGC AAA027-L06 and the genus *Pelagibacter* had ≤2%. Thus, the increase in transporter functions following the rain appears to be directly associated with an increase in the relative proportion of a subset of the organisms that harbor these functions rather than an increase in the distribution of these genes across the community. Organisms with transmembrane transporter genes, especially for organic substrates like organic acids, may be more suited to take advantage of the heterogeneous environment resulting from storm flow conditions.

10.1128/mSphere.00194-18.6 FIG S6  Heat map showing the relative abundance (percentage of total predicted genes) at the level 4 depth of Gene Ontology (GO) terms for the before- and after-rain microbiomes. GO terms that had a higher relative abundance (>100%) in one of the two groups (before versus after rain) compared to the other are shown, and terms that had less than a total of 75 gene counts across all the samples have been excluded from the plot. Samples numbered 1 and 2 for each time point represent biological replicates. Download FIG S6, EPS file, 1.5 MB. Copyright © 2018 Chaudhary et al. 2018 Chaudhary et al. This content is distributed under the terms of the Creative Commons Attribution 4.0 International license.

Additional GOs showing differential abundances included genes related to photosynthesis, biosynthesis of organic compounds such as amines, vitamins, and pigments, as well as the activity of enzyme groups oxidoreductase (acting on the CH-NH_2_ group of donors) and ligase (forming phosphoric ester bonds) that were twice as abundant in the before-rain microbiome (Fig. S6). Genes related to multiorganism processes such as pathogenesis and conjugation were >50% more abundant after rain, while the before-rain microbiome had >50% more functions related to the catabolic process, amine metabolic process, and phosphate-containing compound metabolic process (Fig. 3A). Should the trend of increased pathogenesis and conjugation genes commonly occur with rainfall and persist in the system, it could pose a public health threat, particularly if it promotes the spread of pathogenicity genes throughout the community. Thus, this could be an important group of genes to investigate in future studies.

Further evidence that changes in community composition drove the overall changes in the metabolic capacity came from genes that decreased in relative abundance after rain, such as those encoding biosynthesis of organic substances, which mirrored the overall shifts in taxa (Fig. 2); *Actinobacteria* (39% of the identified sequences at phylum level) and unclassified *Proteobacteria* (31%) were the major taxa encoding organic substance biosynthesis before rain and unclassified *Proteobacteria* (45%) and *Gammaproteobacteria* (13%) after rain. The short-term nature and lack of gene expression data make it difficult to know about the viability and activity of these organisms, but taxon-driven shifts in community functional potential were recently observed in another river in response to sewage and terrestrial-derived organisms (15).

### Biodegradation and antibiotic resistance gene abundance before and after rain.

**FIG 4:**
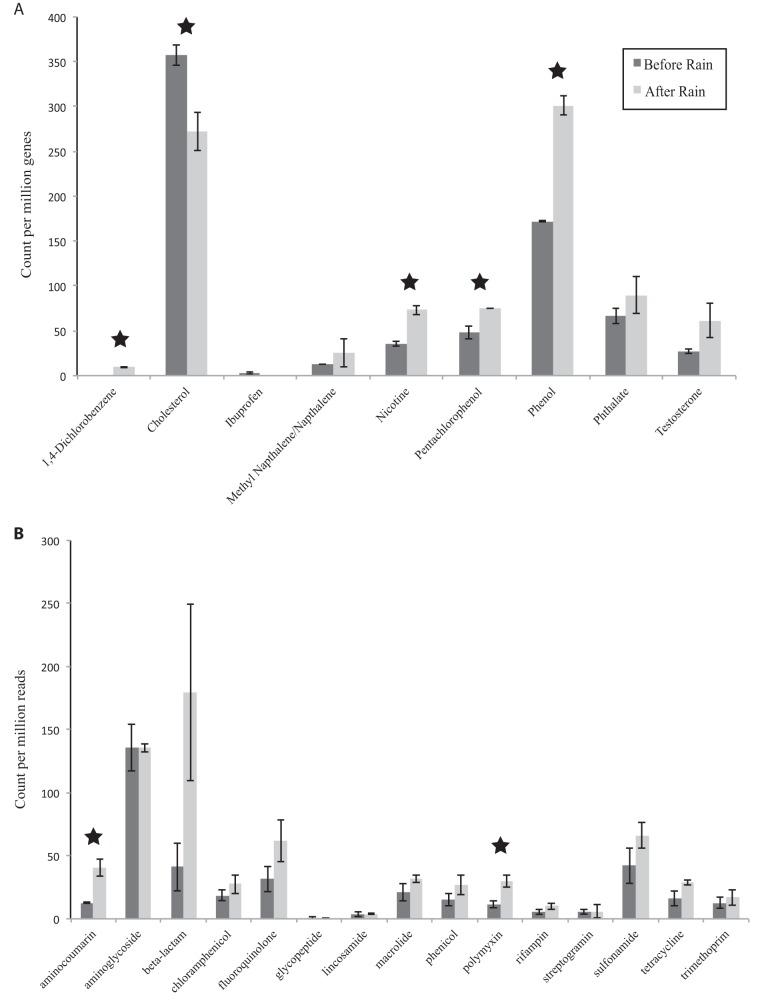
Relative abundance of (A) biodegradation genes (BDGs) and (B) antibiotic resistance genes (ARGs) in the before- and after-rain microbial communities. Relative abundance of BDGs refers to gene count (*in situ* abundance) per million genes per library averaged for each sample for their replicates (*n =* 2) (see Materials and Methods). For ARGs, relative abundance refers to read count per million reads per library averaged for each sample for their replicates. BDGs and ARGs with significant differences in relative abundances between the two time points (*P* < 0.05, *t* test) are highlighted with stars.

In addition to the GO-based functional analysis, we examined how rainfall impacted biodegradation and antibiotic resistance gene content. Predicted open reading frames (ORFs) from both the before- and after-rain metagenomes were searched against a compiled database of protein sequences of microbial enzymes involved in the degradation of 12 different compounds associated with wastewater contamination, stormwater runoff, and WWTP effluent input (Fig. 4A). We detected biodegradation genes (BDGs) in both the before- and after-rain samples for 8 out of the 12 contaminants tested, but observed a significant increase (*P* < 0.05, *t* test) in the relative abundance of genes involved in the degradation of nicotine, phenol, 1,4-dichlorobenzene, and pentachlorophenol and a decrease (*P* < 0.05) in cholesterol-degrading genes after rain (Fig. 4A). Additionally, the total relative abundance of all BDGs was significantly higher in the after-rain sample (*P* < 0.05, *t* test). BDGs before rain were primarily affiliated with unclassified *Proteobacteria* and *Actinobacteria* (35% and 30% of the identified sequences at phylum level, respectively), with the profile shifting to unclassified *Proteobacteria* and *Betaproteobacteria* (49% and 19%, respectively) as the dominant members of the community after rain, similar to the overall taxonomic shifts described above. These results reflect the increase in effluent flow from the WWTP as well as the suspected presence of these compounds in untreated wastewater and CSOs (3, 42–47) (Fig. 4A).

Changes in the relative abundance of antibiotic resistance genes (ARGs) after rain were evaluated using the Comprehensive Antibiotic Resistance Gene Database (CARD). As only a few ORFs (~10 per library) could be classified as ARGs from both the time points, we queried the unassembled paired-end reads against CARD. This resulted in several hits for various ARG categories at both time points (0.04% and 0.07% of the total number of reads for before- and after-rain samples, respectively) and revealed notable increases in the relative abundance of several ARG classes after rain (Fig. 4B), including significant increases in aminocoumarin and polymyxin resistance genes (*P* < 0.05, *t* test). As with the BDGs, the total relative abundance for all ARGs pooled for each time point was significantly higher in the after-rain sample (*P* < 0.05, *t* test). Increases in ARGs with urban-impacted storm flow were recently observed elsewhere as well (14), indicating that this could be a significant and underexplored effect of storm flow. Reads with high matches to ARGs were queried against metagenomic contigs, revealing that unclassified *Proteobacteria* and *Firmicutes* were the abundant ARG-carrying phyla (40% and 23% of the identified sequences at the phylum level, respectively) in the before-rain microbiome, whereas unclassified *Proteobacteria* (50%) and *Gammaproteobacteria* (24%) were the dominant groups after the rain. This further supports the importance of taxon-driven changes on gene content.

The results for both community composition and functional gene analysis provide evidence for the significant influence of storm flow-related input on the microbial community, particularly from increased WWTP effluent flow rates associated with heavy rain. Overall, this study revealed a shift in microbial community composition following rain from organisms frequently associated with freshwater systems toward organisms associated with urban-impacted waters (9, 19, 20), as well as a shift in functional gene content. The increased relative abundance (and possibly actual abundance) of BDGs and ARGs along with the increase in genes associated with conjugation and pathogenesis in the after rain microbiome highlight the environmental and public health implications of storm flow in urban waterways. The extent to which these changes in gene content are expressed metabolically and persist is unknown. Although the WGS metagenomic analysis of a single rainfall event limits the scope of interpretations that can be drawn, our results provide substantial insights into microbial community dynamics in an urban stream during storm flow conditions, highlighting the need to investigate the urban stream microbiome with longer temporal scales and systematic sampling design to better predict the impact of rain-associated storm flow events.

## MATERIALS AND METHODS

### Site description and sample collection.

The North Shore Channel (NSC) is a 12.3-km-long man-made stream of the Chicago Area Waterway System that receives freshwater input from Lake Michigan and effluent input from the O’Brien Water Reclamation Plant, a WWTP that serves over 1.3 million people residing in a 365-km^2^ area (http://www.mwrd.org/irj/portal/anonymous/waterreclamation). Our study site is approximately 1 km downstream of the WWTP outfall (Fig. S1). The NSC also has 48 CSOs along its course, six of which are located within about 1 km upstream of WWTP, and two of which are located within 1 km downstream of the WWTP. These release excess stormwater mixed with untreated sewage into the river when the transport and storage capacity of the city’s sewage network is exceeded following high rainfall (http://www.mwrd.org/irj/portal/anonymous/overview) (Fig. S1). Water from the selected NSC site was sampled five times between 2013 and 2015 (0- to 1-m depth): three samplings represent stream water during base flow (dry weather) conditions, and the other two represent storm flow (<24 h after rainfall) conditions (details are in Table S1). We also sampled the WWTP effluent in October 2013 during base flow conditions. Additional sample metadata and water chemistry are given in Table S1.

Water was collected using a horizontal sampler (Wildco, Yulee, FL) and passed on-site in succession through ~1.6-µm-pore-size glass fiber filters to remove larger particles (Whatman, Pittsburgh, PA), and cells were collected on 0.22-µm-pore-size polycarbonate membrane filters (EMD Millipore, Billerica, MA). WWTP effluent was collected from the WWTP outlet where the released effluent mixes with stream water. About 10 liters of water was filtered in duplicate for each NSC sampled time point (for effluent, a single ~10-liter sample was obtained), and ~20 ml of the filtrate was transported back to the lab for chemical analysis. Water temperature, pH, conductivity, and total dissolved solids were measured on-site using a portable water quality meter (Hanna Instruments, Woonsocket, RI). Additional water chemistry analysis is described in Table S1.

### DNA extraction and sequencing.

DNA was extracted from filters as described in reference 48. Briefly, filters were incubated in lysis buffer (50 mM Tris-HCl, 40 mM EDTA, 0.75 M sucrose) containing 1 mg/ml lysozyme and 200 µg/ml RNase at 37°C for 30 min. Subsequently, the samples were incubated with 1% SDS and 10 mg/ml proteinase K at 55°C and rotated overnight. From the lysate, DNA was extracted using phenol-chloroform, followed by ethanol precipitation and elution in Tris-EDTA (TE) buffer.

Whole-genome shotgun (WGS) metagenomic sequencing was done on the Illumina HiSeq (v1) with a paired-end format and a read length of 150 bp at the Michigan State University Research Technology Support Facility. We obtained 2.82 and 3.18 Gbp of paired-end read data for the before- and after-rain samples, respectively. Replicate filters were sequenced at the University of Illinois at the Chicago DNA Services Facility (DNAS) on a single lane of the Illumina HiSeq platform with paired-end format and read length of 100 bp, yielding 4.04 and 1.31 Gbp of paired-end read data for the before- and after-rain libraries, respectively.

For 16S rRNA gene amplicon sequencing, 10 to 30 ng of DNA from each biological replicate (filter) was amplified with the V1 to V3 primers 27F and 534R (49, 50). Amplicons were sequenced at the DNAS on the Illumina MiSeq platform with the paired-end format and read length of 300 bp. Between 28,933 and 160,811 sequences per sample were obtained, with an average of 61,337 sequences per sample.

### 16S rRNA gene-based analysis of microbial community diversity.

Paired-end bar-coded reads of 16S rRNA gene amplicons were obtained for all the time points sampled and quality filtered using Trimmomatic (51), with a minimum average quality score of 20 across a 4-base sliding window and a minimum read length of 100 bp (including primer) posttrimming. Trimmed, paired-end reads were merged using Pear (52), but due to low yield of the merged reads, likely due to issues related to the MiSeq V2 kit chemistry, further analysis was only performed on the trimmed forward reads. Reads were analyzed using QIIME version 1.8.0 (53). Library statistics are summarized in Table S2. Chimeric sequences were removed using *identify_chimeric_seqs.py* with the usearch61 denovo method and *filter_fasta.py*. Filtered sequences were clustered into operational taxonomic units (OTUs) at a 97% identity level using scripts *pick_otus.py* and *pick_rep_set.py* based on usearch61 denovo OTU picking. Representative OTUs were assigned taxonomy based on the Greengenes reference database (May 2013 version) using *assign_taxonomy.py* with uclust. OTUs occurring as singletons or with sequences from just one library were excluded from analyses. Determination of community taxonomic composition and alpha diversity was performed using *summarize_taxa.py* and *alpha_diversity.py*, respectively, with a random subsample of 17,384 sequences per sample to avoid bias arising from variation in sequencing depth. Good’s coverage for each library was estimated using *alpha_diversity.py* and OTUs that included singletons, subsampled to an even depth of 18,289 sequences per library, the smallest library size.

### Metagenomic sequence assembly and phylogenetic classification.

Raw metagenomic sequences were quality filtered using a Phred average per sliding window with a quality threshold (*Q*) of ≥20 and not allowing any *N* values. Quality-filtered coupled reads for each metagenomic library were assembled as described in reference 48. Coupled reads were first assembled into contigs with Velvet (54) and SOAPdenovo2 (55) separately and input to Newbler 2.0 to obtain longer contigs with better *N*_50_ values (56). Additional metagenomic library statistics are provided in Table S3. Gene calling was done with MetaGeneMark (57). Due to uneven data yields from sequencing, we used assemblies from the first sequencing run for each sample as the representative sequences for annotations and mapped the coupled reads from both the replicate libraries to these contigs for each sample to calculate the contig coverage in each library. The predicted protein-coding genes for each data set were used for phylogenetic classification of the corresponding contigs using MyTaxa (28) with a database of all sequenced bacterial and archaeal genomes (http://enve-omics.ce.gatech.edu/data/mytaxa) using DIAMOND blastp in the sensitive mode (58). Reads were mapped to contigs using blastn with cutoffs of ≥50% alignment length, identity of ≥97%, and an E value of ≤10^−10^. Contig coverage (sum of lengths of reads mapping to contig/contig length) was used as a proxy for *in situ* abundance in each library and calculated using the *BlastTab.seqdepth_nomedian.pl* script from the Enveomics bioinformatics toolbox (59). The script aai.rb from the same toolbox was used to calculate average amino acid identity (AAI) between any two sets of protein-coding genes.

### Analysis of functional gene content and antibiotic resistance genes.

Predicted metagenomic genes were searched against the Swiss-Prot database (60) using blastp and cutoffs of at least 40% sequence identity, 70% coverage of the query sequence, and an E value of ≤10^−10^. The Swiss-Prot match for the best hit for each query sequence was mapped to its corresponding Gene Ontology (GO) term (61), followed by binning the characterized genes at various depths (distance of a GO term from the parent node) of the GO database using the Semantics collection of scripts in the Enveomics toolbox (http://enveomics.blogspot.com/2012/11/semantics.html). To evaluate the functional profile at a specific depth, *in situ* abundance for these GO terms was calculated using gene coverage (described above), and relative abundance for each GO term was obtained as a fraction of the total abundance of genes with identified functions in that library. The taxonomic affiliation of genes classified within a specific GO term was evaluated using MyTaxa, as described above.

To specifically evaluate the presence and abundance of genes involved in biodegradation of select wastewater contaminants in the rain-associated metagenomes, we created a database of protein sequences of enzymes related to degradation of select contaminants that are commonly found in WWTP effluent and sewage: testosterone, ibuprofen, caffeine, nicotine, cholesterol, 1,4-dichlorobenzene, methylnaphthalene, pentachlorophenol, phenol, *N*,*N*-diethyl-3-toluamide, tetrachloroethylene, and phthalate (3, 42–47). The enzymes were selected based on their role in the degradation pathways for these compounds (62), as well as the sequence availability in NCBI. This database is available from the corresponding author upon request. The predicted ORFs were searched against this database using blastp, and the best hits were filtered at same thresholds used for Swiss-Prot (described above). Coverage estimates were used for calculation of the *in situ* abundance for each BDG class and normalized for each library by dividing the abundance of each BDG class by the total coverage of all predicted genes in that library and multiplying the result by 1 million to obtain gene count per million genes per library.

Antibiotic resistance genes in the rain-associated samples were identified by searching the predicted ORFs as well as paired-end metagenomic reads against the Comprehensive Antibiotic Resistance Gene Database (CARD) (63) using blastp and blastx and a threshold of at least 80% sequence identity and 80% coverage of the query sequence (64, 65). Filtered reads for each library were binned into broad antibiotic resistance categories using the Resistance Gene Categories index file provided on the CARD website (http://arpcard.mcmaster.ca/), and the read counts for each category were normalized for the library size as read count for ARG category per million reads per library.

### Microbial abundance estimation using fluorescence microscopy.

October 2013 NSC samples were fixed with paraformaldehyde (1% final concentration) in triplicate and stored in 4°C. Samples were then vortexed and collected on 25-mm black polycarbonate filters (0.2-µm-pore size) and stained with 5 µl of a 10-mg/ml DAPI (4′,6-diamidino-2-phenylindole) working solution diluted in 10× phosphate-buffered saline (PBS). Microbial cells were enumerated (three slides from three replicate samples per time point) with an epifluorescence microscope (Zeiss Axio Scope.A1).

### Statistical analyses.

Analysis of similarity (ANOSIM) and similarity percentage (SIMPER) analysis on 16S rRNA gene and metagenomic community composition data sets, respectively, were performed using the R vegan package (66). The Statistical Analysis of Metagenomic Profiles (STAMP) software package was used for two-tailed Student’s *t* tests or Welch’s *t* tests to evaluate differentially abundant taxonomic groups among the 16S rRNA gene and metagenomic data sets (67) (multiple test correction, if applied, was done using Storey's false-discovery rate correction), and R was used for these tests to evaluate differentially abundant physicochemical parameters, ARGs, and BDGs. Principal-coordinate analysis (PCoA; Bray-Curtis metric) of OTUs (with singletons removed and the table subsampled to an even depth per sample) was performed with the Phyloseq package in R (68).

### Accession number(s).

All of the sequence data in this study have been submitted to the Sequence Read Archive at NCBI under accession no. SRP080963.

10.1128/mSphere.00194-18.10 TABLE S4  Rare species in the before-rain microbiome that were in the abundant fraction after rain. Download TABLE S4, DOCX file, 0.1 MB. Copyright © 2018 Chaudhary et al. 2018 Chaudhary et al. This content is distributed under the terms of the Creative Commons Attribution 4.0 International license.

## References

[1] Cole JJ, Prairie YT, Caraco NF, Mcdowell WH, Tranvik LJ, Striegl RG, Duarte CM, Kortelainen P, Downing JA, Middelburg JJ, Melack J 2007 Plumbing the global carbon cycle: integrating inland waters into the terrestrial carbon budget. Ecosystems 10:172–185. doi:10.1007/s10021-006-9013-8.

[2] Paul MJ, Meyer JL 2001 Streams in the urban landscape. Annu Rev Ecol Syst 32:333–365. doi:10.1146/annurev.ecolsys.32.081501.114040.

[3] Phillips PJ, Chalmers AT, Gray JL, Kolpin DW, Foreman WT, Wall GR 2012 Combined sewer overflows: an environmental source of hormones and wastewater micropollutants. Environ Sci Technol 46:5336–5343. doi:10.1021/es3001294. 22540536 PMC3352270

[4] Weyrauch P, Matzinger A, Pawlowsky-Reusing E, Plume S, von Seggern D, Heinzmann B, Schroeder K, Rouault P 2010 Contribution of combined sewer overflows to trace contaminant loads in urban streams. Water Res 44:4451–4462. doi:10.1016/j.watres.2010.06.011. 20599243

[5] Walsh CJ, Roy AH, Feminella JW, Cottingham PD, Groffman PM, Morgan RP 2005 The urban stream syndrome: current knowledge and the search for a cure. J North Am Benthol Soc 24:706–723. doi:10.1899/04-028.1.

[6] Rechenburg A, Koch C, Classen T, Kistemann T 2006 Impact of sewage treatment plants and combined sewer overflow basins on the microbiological quality of surface water. Water Sci Technol 54:95–99. doi:10.2166/wst.2006.454. 17037139

[7] Sercu B, Van De Werfhorst LC, Murray J, Holden PA 2009 Storm drains are sources of human fecal pollution during dry weather in three urban Southern California watersheds. Environ Sci Technol 43:293–298. doi:10.1021/es801505p. 19238954

[8] Newton RJ, Bootsma MJ, Morrison HG, Sogin ML, McLellan SL 2013 A microbial signature approach to identify fecal pollution in the waters off an urbanized coast of Lake Michigan. Microb Ecol 65:1011–1023. doi:10.1007/s00248-013-0200-9. 23475306 PMC4084971

[9] Fisher JC, Newton RJ, Dila DK, McLellan SL 2015 Urban microbial ecology of a freshwater estuary of Lake Michigan. Elementa 3:000064. doi:10.12952/journal.elementa.000064. PMC4746012 26866046

[10] Drury B, Rosi-Marshall E, Kelly JJ 2013 Wastewater treatment effluent reduces the abundance and diversity of benthic bacterial communities in urban and suburban rivers. Appl Environ Microbiol 79:1897–1905. doi:10.1128/AEM.03527-12. 23315724 PMC3592216

[11] Drury B, Scott J, Rosi-Marshall EJ, Kelly JJ 2013 Triclosan exposure increases triclosan resistance and influences taxonomic composition of benthic bacterial communities. Environ Sci Technol 47:8923–8930. doi:10.1021/es401919k. 23865377

[12] Czekalski N, Berthold T, Caucci S, Egli A, Bürgmann H 2012 Increased levels of multiresistant bacteria and resistance genes after wastewater treatment and their dissemination into Lake Geneva, Switzerland. Front Microbiol 3:106. doi:10.3389/fmicb.2012.00106. 22461783 PMC3310248

[13] Rizzo L, Manaia C, Merlin C, Schwartz T, Dagot C, Ploy MC, Michael I, Fatta-Kassinos D 2013 Urban wastewater treatment plants as hotspots for antibiotic resistant bacteria and genes spread into the environment: a review. Sci Total Environ 447:345–360. doi:10.1016/j.scitotenv.2013.01.032. 23396083

[14] Zhang S, Pang S, Wang PF, Wang C, Han N, Liu B, Han B, Li Y, Anim-Larbi K 2016 Antibiotic concentration and antibiotic-resistant bacteria in two shallow urban lakes after stormwater event. Environ Sci Pollut Res 23:9984–9992. doi:10.1007/s11356-016-6237-9. 26865482

[15] Meziti A, Tsementzi D, Ar Kormas K, Karayanni H, Konstantinidis KT 2016 Anthropogenic effects on bacterial diversity and function along a river-to-estuary gradient in Northwest Greece revealed by metagenomics. Environ Microbiol 18:4640–4652. doi:10.1111/1462-2920.13303. 27001690

[16] Jeffries TC, Schmitz Fontes ML, Harrison DP, Van-Dongen-Vogels V, Eyre BD, Ralph PJ, Seymour JR 2016 Bacterioplankton dynamics within a large anthropogenically impacted urban estuary. Front Microbiol 6:1438. doi:10.3389/fmicb.2015.01438. 26858690 PMC4726783

[17] Newton RJ, Jones SE, Eiler A, McMahon KD, Bertilsson S 2011 A guide to the natural history of freshwater lake bacteria. Microbiol Mol Biol Rev 75:14–49. doi:10.1128/MMBR.00028-10. 21372319 PMC3063352

[18] Ghai R, Mizuno CM, Picazo A, Camacho A, Rodriguez-Valera F 2014 Key roles for freshwater Actinobacteria revealed by deep metagenomic sequencing. Mol Ecol 23:6073–6090. doi:10.1111/mec.12985. 25355242

[19] Newton RJ, McLellan SL 2015 A unique assemblage of cosmopolitan freshwater bacteria and higher community diversity differentiate an urbanized estuary from oligotrophic Lake Michigan. Front Microbiol 6:1028. doi:10.3389/fmicb.2015.01028. 26483766 PMC4586452

[20] McLellan SL, Fisher JC, Newton RJ 2015 The microbiome of urban waters. Int Microbiol 18:141–149. doi:10.2436/20.1501.01.244. 27036741 PMC8793681

[21] Rodriguez-R LM, Konstantinidis KT 2014 Nonpareil: a redundancy-based approach to assess the level of coverage in metagenomic datasets. Bioinformatics 30:629–635. doi:10.1093/bioinformatics/btt584. 24123672

[22] Kirs M, Kisand V, Wong M, Caffaro-Filho RA, Moravcik P, Harwood VJ, Yoneyama B, Fujioka RS 2017 Multiple lines of evidence to identify sewage as the cause of water quality impairment in an urbanized tropical watershed. Water Res 116:23–33. doi:10.1016/j.watres.2017.03.024. 28292677

[23] Illinois Department of Natural Resources 2011 Illinois Coastal Management Program issue paper: Chicago River and North Shore Channel corridors. Illinois Department of Natural Resources, Springfield, IL.

[24] Poretsky R, Rodriguez-R LM, Luo C, Tsementzi D, Konstantinidis KT 2014 Strengths and limitations of 16S rRNA gene amplicon sequencing in revealing temporal microbial community dynamics. PLoS One 9:e93827. doi:10.1371/journal.pone.0093827. 24714158 PMC3979728

[25] Clarke KR 1993 Non-parametric multivariate analyses of changes in community structure. Aust Ecol 18:117–143. doi:10.1111/j.1442-9993.1993.tb00438.x.

[26] Kingry LC, Petersen JM 2014 Comparative review of Francisella tularensis and Francisella novicida. Front Cell Infect Microbiol 4:35. doi:10.3389/fcimb.2014.00035. 24660164 PMC3952080

[27] Stratton CW, Mitchell WM 1996 The pathogenesis of *Chlamydia* species. Antimicrob Infect Dis Newsl 15:83–88. doi:10.1016/S1069-417X(01)80014-5.

[28] Luo C, Rodriguez-R LM, Konstantinidis KT 2014 MyTaxa: an advanced taxonomic classifier for genomic and metagenomic sequences. Nucleic Acids Res 42:e73. doi:10.1093/nar/gku169. 24589583 PMC4005636

[29] Rodriguez-R LM, Konstantinidis KT 2014 Bypassing cultivation to identify bacterial species. Microbe 9:111–118. doi:10.1128/microbe.9.111.1.

[30] Lee HK, Shim JI, Kim HE, Yu JY, Kang YH 2010 Distribution of *Legionella* species from environmental water sources of public facilities and genetic diversity of L. pneumophila serogroup 1 in South Korea. Appl Environ Microbiol 76:6547–6554. doi:10.1128/AEM.00422-10. 20693456 PMC2950455

[31] Bartram J, Chartier Y, Lee JV, Pond K, Surman-Lee S (ed) 2007 Legionella and the prevention of legionellosis. WHO, Geneva, Switzerland.

[32] Petersen JM, Mead PS, Schriefer ME 2009 *Francisella tularensis*: an arthropod-borne pathogen. Vet Res 40:7. doi:10.1051/vetres:2008045. 18950590 PMC2695023

[33] Garcia SL, McMahon KD, Martinez-Garcia M, Srivastava A, Sczyrba A, Stepanauskas R, Grossart HP, Woyke T, Warnecke F 2013 Metabolic potential of a single cell belonging to one of the most abundant lineages in freshwater bacterioplankton. ISME J 7:137–147. doi:10.1038/ismej.2012.86. 22810059 PMC3526179

[34] Ghai R, Rodriguez-Valera F, McMahon KD, Toyama D, Rinke R, Cristina Souza de Oliveira T, Wagner Garcia J, Pellon de Miranda F, Henrique-Silva F 2011 Metagenomics of the water column in the pristine upper course of the Amazon River. PLoS One 6:e23785. doi:10.1371/journal.pone.0023785. 21915244 PMC3158796

[35] Warnecke F, Amann R, Pernthaler J 2004 Actinobacterial 16S rRNA genes from freshwater habitats cluster in four distinct lineages. Environ Microbiol 6:242–253. doi:10.1111/j.1462-2920.2004.00561.x. 14871208

[36] Satinsky BM, Fortunato CS, Doherty M, Smith CB, Sharma S, Ward ND, Krusche AV, Yager PL, Richey JE, Moran MA, Crump BC 2015 Metagenomic and metatranscriptomic inventories of the lower Amazon River, May 2011. Microbiome 3:39. doi:10.1186/s40168-015-0099-0. 26353777 PMC4564970

[37] Ghylin TW, Garcia SL, Moya F, Oyserman BO, Schwientek P, Forest KT, Mutschler J, Dwulit-Smith J, Chan LK, Martinez-Garcia M, Sczyrba A, Stepanauskas R, Grossart HP, Woyke T, Warnecke F, Malmstrom R, Bertilsson S, McMahon KD 2014 Comparative single-cell genomics reveals potential ecological niches for the freshwater acI Actinobacteria lineage. ISME J 8:2503–2516. doi:10.1038/ismej.2014.135. 25093637 PMC4260696

[38] Poretsky RS, Sun S, Mou X, Moran MA 2010 Transporter genes expressed by coastal bacterioplankton in response to dissolved organic carbon. Environ Microbiol 12:616–627. doi:10.1111/j.1462-2920.2009.02102.x. 19930445 PMC2847192

[39] Rinta-Kanto JM, Sun S, Sharma S, Kiene RP, Moran MA 2012 Bacterial community transcription patterns during a marine phytoplankton bloom. Environ Microbiol 14:228–239. doi:10.1111/j.1462-2920.2011.02602.x. 21985473

[40] Pitt FD, Mazard S, Humphreys L, Scanlan DJ 2010 Functional characterization of Synechocystis sp. strain PCC 6803 pst1 and pst2 gene clusters reveals a novel strategy for phosphate uptake in a freshwater cyanobacterium. J Bacteriol 192:3512–3523. doi:10.1128/JB.00258-10. 20435726 PMC2897655

[41] Sangwan N, Zarraonaindia I, Hampton-Marcell JT, Ssegane H, Eshoo TW, Rijal G, Negri MC, Gilbert JA 2016 Differential functional constraints cause strain-level endemism in *Polynucleobacter* populations. mSystems 1:e00003-16. doi:10.1128/mSystems.00003-16. PMC5069759 27822527

[42] Boyd GR, Palmeri JM, Zhang S, Grimm DA 2004 Pharmaceuticals and personal care products (PPCPs) and endocrine disrupting chemicals (EDCs) in stormwater canals and Bayou St. John in New Orleans, Louisiana, USA. Sci Total Environ 333:137–148. doi:10.1016/j.scitotenv.2004.03.018. 15364525

[43] Glassmeyer ST, Furlong ET, Kolpin DW, Cahill JD, Zaugg SD, Werner SL, Meyer MT, Kryak DD 2005 Transport of chemical and microbial compounds from known wastewater discharges: potential for use as indicators of human fecal contamination. Environ Sci Technol 39:5157–5169. doi:10.1021/es048120k. 16082943

[44] Benotti MJ, Brownawell BJ 2007 Distributions of pharmaceuticals in an urban estuary during both dry- and wet-weather conditions. Environ Sci Technol 41:5795–5802. doi:10.1021/es0629965. 17874789

[45] Phillips P, Chalmers A 2009 Wastewater effluent, combined sewer overflows, and other sources of organic compounds to Lake Champlain. J Am Water Resour Assoc 45:45–57. doi:10.1111/j.1752-1688.2008.00288.x.

[46] Sauvé S, Aboulfadl K, Dorner S, Payment P, Deschamps G, Prévost M 2012 Fecal coliforms, caffeine and carbamazepine in stormwater collection systems in a large urban area. Chemosphere 86:118–123. doi:10.1016/j.chemosphere.2011.09.033. 22075053

[47] Fang H, Cai L, Yu Y, Zhang T 2013 Metagenomic analysis reveals the prevalence of biodegradation genes for organic pollutants in activated sludge. Bioresour Technol 129:209–218. doi:10.1016/j.biortech.2012.11.054. 23247148

[48] Oh S, Caro-Quintero A, Tsementzi D, DeLeon-Rodriguez N, Luo C, Poretsky R, Konstantinidis KT 2011 Metagenomic insights into the evolution, function, and complexity of the planktonic microbial community of Lake Lanier, a temperate freshwater ecosystem. Appl Environ Microbiol 77:6000–6011. doi:10.1128/AEM.00107-11. 21764968 PMC3165412

[49] Frank JA, Reich CI, Sharma S, Weisbaum JS, Wilson BA, Olsen GJ 2008 Critical evaluation of two primers commonly used for amplification of bacterial 16S rRNA genes. Appl Environ Microbiol 74:2461–2470. doi:10.1128/AEM.02272-07. 18296538 PMC2293150

[50] Somenahally AC, Mosher JJ, Yuan T, Podar M, Phelps TJ, Brown SD, Yang ZK, Hazen TC, Arkin AP, Palumbo AV, Van Nostrand JD, Zhou J, Elias DA 2013 Hexavalent chromium reduction under fermentative conditions with lactate stimulated native microbial communities. PLoS One 8:e83909. doi:10.1371/journal.pone.0083909. 24376771 PMC3871698

[51] Bolger AM, Lohse M, Usadel B 2014 Trimmomatic: a flexible trimmer for Illumina sequence data. Bioinformatics 30:2114–2120. doi:10.1093/bioinformatics/btu170. 24695404 PMC4103590

[52] Zhang J, Kobert K, Flouri T, Stamatakis A 2014 PEAR: a fast and accurate Illumina Paired-End reAd mergeR. Bioinformatics 30:614–620. doi:10.1093/bioinformatics/btt593. 24142950 PMC3933873

[53] Caporaso JG, Kuczynski J, Stombaugh J, Bittinger K, Bushman FD, Costello EK, Fierer N, Peña AG, Goodrich JK, Gordon JI, Huttley GA, Kelley ST, Knights D, Koenig JE, Ley RE, Lozupone CA, McDonald D, Muegge BD, Pirrung M, Reeder J, Sevinsky JR, Turnbaugh PJ, Walters WA, Widmann J, Yatsunenko T, Zaneveld J, Knight R 2010 QIIME allows analysis of high-throughput community sequencing data. Nat Methods 7:335–336. doi:10.1038/nmeth.f.303. 20383131 PMC3156573

[54] Zerbino DR, Birney E 2008 Velvet: algorithms for de novo short read assembly using de Bruijn graphs. Genome Res 18:821–829. doi:10.1101/gr.074492.107. 18349386 PMC2336801

[55] Luo R, Liu B, Xie Y, Li Z, Huang W, Yuan J, He G, Chen Y, Pan Q, Liu Y, Tang J, Wu G, Zhang H, Shi Y, Liu Y, Yu C, Wang B, Lu Y, Han C, Cheung DW, Yiu SM, Peng S, Xiaoqian Z, Liu G, Liao X, Li Y, Yang H, Wang J, Lam TW, Wang J 2012 SOAPdenovo2: an empirically improved memory-efficient short-read de novo assembler. Gigascience 1:18. doi:10.1186/2047-217X-1-18. 23587118 PMC3626529

[56] Luo C, Tsementzi D, Kyrpides NC, Konstantinidis KT 2012 Individual genome assembly from complex community short-read metagenomic datasets. ISME J 6:898–901. doi:10.1038/ismej.2011.147. 22030673 PMC3309356

[57] Zhu W, Lomsadze A, Borodovsky M 2010 Ab initio gene identification in metagenomic sequences. Nucleic Acids Res 38:e132. doi:10.1093/nar/gkq275. 20403810 PMC2896542

[58] Buchfink B, Xie C, Huson DH 2015 Fast and sensitive protein alignment using DIAMOND. Nat Methods 12:59–60. doi:10.1038/nmeth.3176. 25402007

[59] Rodriguez-R LM, Konstantinidis KT 2016 The enveomics collection: a toolbox for specialized analyses of microbial genomes and metagenomes. Peer J Prepr 4:e1900 v1 10.7287/peerj.preprints.1900v1.

[60] Wu CH, Apweiler R, Bairoch A, Natale DA, Barker WC, Boeckmann B, Ferro S, Gasteiger E, Huang H, Lopez R, Magrane M, Martin MJ, Mazumder R, O’Donovan C, Redaschi N, Suzek B 2006 The Universal Protein Resource (UniProt): an expanding universe of protein information. Nucleic Acids Res 34:D187–D191. doi:10.1093/nar/gkj161. 16381842 PMC1347523

[61] Ashburner M, Ball CA, Blake JA, Botstein D, Butler H, Cherry JM, Davis AP, Dolinski K, Dwight SS, Eppig JT, Harris MA, Hill DP, Issel-Tarver L, Kasarskis A, Lewis S, Matese JC, Richardson JE, Ringwald M, Rubin GM, Sherlock G 2000 Gene Ontology: tool for the unification of biology. The Gene Ontology Consortium. Nat Genet 25:25–29. doi:10.1038/75556. 10802651 PMC3037419

[62] Gao J, Ellis LBM, Wackett LP 2010 The University of Minnesota Biocatalysis/Biodegradation Database: improving public access. Nucleic Acids Res 38:D488–D491. doi:10.1093/nar/gkp771. 19767608 PMC2808978

[63] McArthur AG, Waglechner N, Nizam F, Yan A, Azad MA, Baylay AJ, Bhullar K, Canova MJ, De Pascale G, Ejim L, Kalan L, King AM, Koteva K, Morar M, Mulvey MR, O’Brien JS, Pawlowski AC, Piddock LJV, Spanogiannopoulos P, Sutherland AD, Tang I, Taylor PL, Thaker M, Wang W, Yan M, Yu T, Wright GD 2013 The comprehensive antibiotic resistance database. Antimicrob Agents Chemother 57:3348–3357. doi:10.1128/AAC.00419-13. 23650175 PMC3697360

[64] Gibson MK, Forsberg KJ, Dantas G 2015 Improved annotation of antibiotic resistance determinants reveals microbial resistomes cluster by ecology. ISME J 9:207–216. doi:10.1038/ismej.2014.106. 25003965 PMC4274418

[65] Port JA, Cullen AC, Wallace JC, Smith MN, Faustman EM 2014 Metagenomic frameworks for monitoring antibiotic resistance in aquatic environments. Environ Health Perspect 122:222–228. doi:10.1289/ehp.1307009. 24334622 PMC3948035

[66] Oksanen J, Blanchet FG, Kindt R, Legendre P, Minchin PR, O’Hara RB, Simpson GL, Solymos P, Stevens MHH, Wagner H 2015 vegan: Community Ecology package. R package version 2.2-1. R Foundation for Statistical Computing, Vienna, Austria http://CRANR-project.org/package=vegan.

[67] Parks DH, Tyson GW, Hugenholtz P, Beiko RG 2014 STAMP: statistical analysis of taxonomic and functional profiles. Bioinformatics 30:3123–3124. doi:10.1093/bioinformatics/btu494. 25061070 PMC4609014

[68] McMurdie PJ, Holmes S 2013 Phyloseq: an R package for reproducible interactive analysis and graphics of microbiome census data. PLoS One 8:e61217. doi:10.1371/journal.pone.0061217. 23630581 PMC3632530

