# Supplementary figures and images for: Taxon-Driven Functional Shifts Associated with Storm Flow in an Urban Stream Microbial Community
Source: mSphere. 2018 Jul 5;3(4):e00194-18. doi: 10.1128/mSphere.00194-18 (PMC6034075; doi:10.1128/mSphere.00194-18)

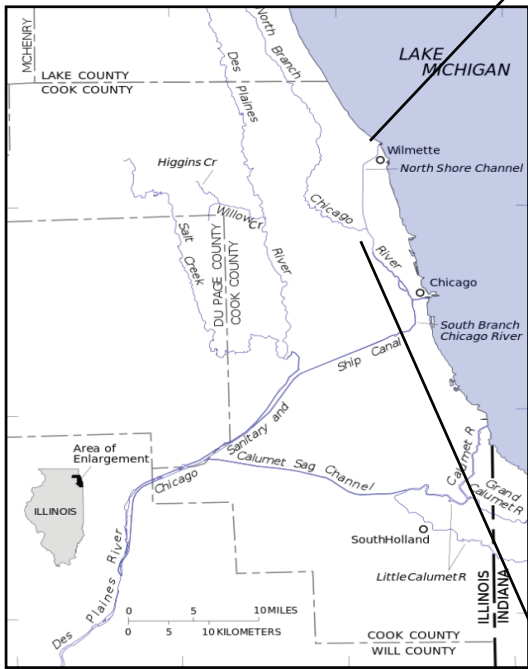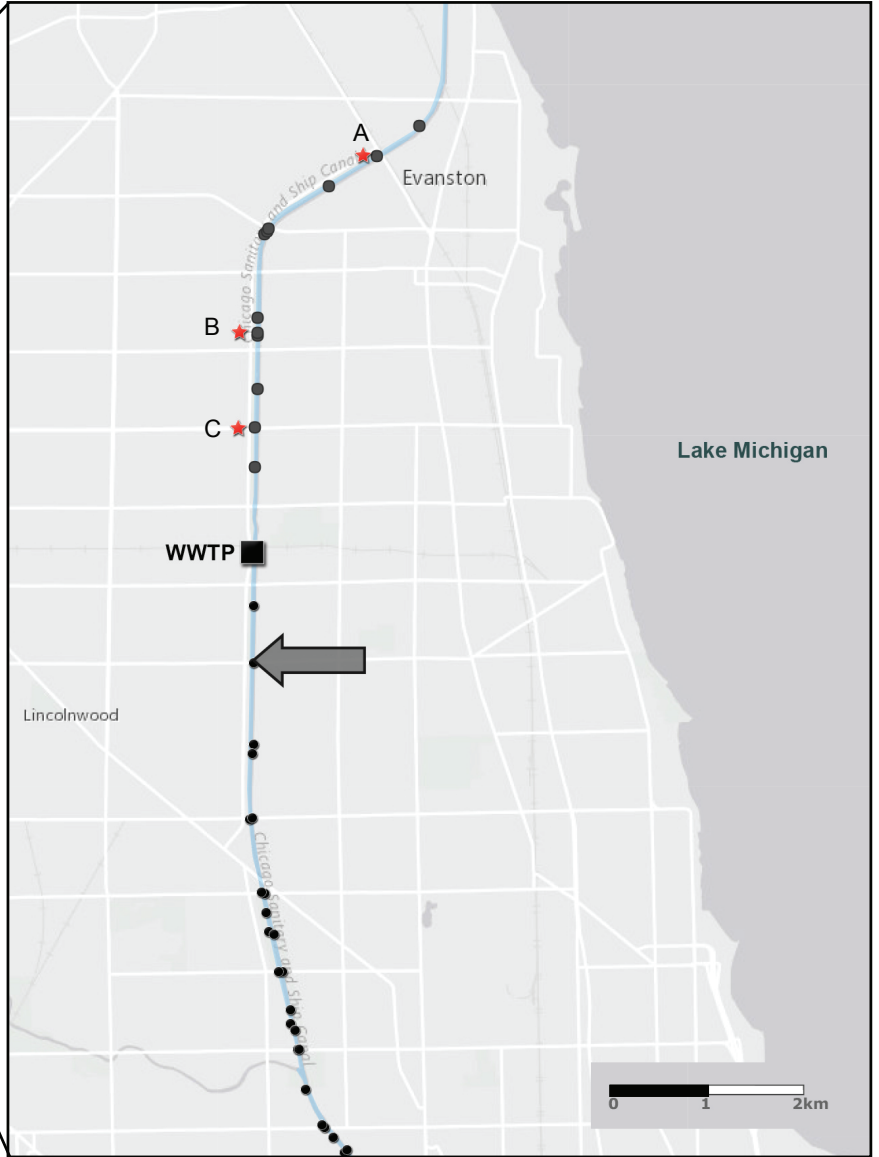

Supplement: FIG S1 [file sph004182588sf1.pdf]
