# Supplementary material for: Taxon-Driven Functional Shifts Associated with Storm Flow in an Urban Stream Microbial Community
Source: mSphere. 2018 Jul 5;3(4):e00194-18. doi: 10.1128/mSphere.00194-18 (PMC6034075; doi:10.1128/mSphere.00194-18)
Supplement: TABLE S1 [file sph004182588st1.docx]

| Sample ID | Sampling  Date | Weather^a^ | SRP^b,c^ (mg P/L) | NH_3_ /NH_4_^+c^  (mg N/L) | NO_3_^-c^  (mg N/L) | TDS^d^ (ppm) | pH | C^d^ (μS/cm) | T^d^ (°C) |
| --- | --- | --- | --- | --- | --- | --- | --- | --- | --- |
| Before Rain  Oct. 2013 | 10/05/2013 | Baseflow | 0.80 ± 0.09 | 0.41 ± 0.03 | 5.74 ± 0.56 | 286 | 7.6 | 566 | 22.7 |
| After Rain  Oct. 2013 | 10/06/2013 | Stormflow | 0.49 ± 0.02 | 0.26 ± 0.01 | 6.87 ± 0.06 | 308 | 7.5 | 604 | 18.5 |
| July 2014 | 07/08/2014 | Stormflow | 0.56 ± 0.00 | 0.35 ± 0.00 | 5.11 ± 0.17 | 413 | 7.0 | 813 | 24 |
| Oct. 2014 | 10/25/2014 | Baseflow | 1.82 ± 0.10 | 1.04 ± 0.00 | 9.62 ± 0.08 | 471 | 7.1 | 923 | 20.5 |
| July 2015 | 07/23/2015 | Baseflow | NA^e^ | NA^e^ | NA^e^ | 324 | 7.5 | 675 | 24.5 |
| Effluent.  Oct. 2013 | 10/05/2013  (WWTP Effluent) | Baseflow | 1.5^f^ | 0.3^f^ | 10.93^f^ | 360 | 7.4 | 707 | 23.2 |

^a^ Weather : ‘Baseflow’ condition represents no rainfall event (<2.5 mm precipitation) for at least 72 h prior to sample collection, and ‘stormflow’ represents sample collection <24 h after rainfall (>10 mm precipitation)

^b^ SRP: Soluble Reactive Phosphate

^c^ The concentrations for SRP, NH_3_ and NO_3_^-^ are mean values for duplicate samples with their standard errors. NH_3_ or NH_4_^+^ was measured as per the methodology (Hach kits/AutoAnalyzer 3)

^d^ TDS: Total Dissolved Solids; C: Conductivity; T: Temperature

^e^ NA: Data not available

^f^ MWRD data for the O’Brien Water Reclamation Plant effluent for the date sampled

October 2013 rain-associated filtrate samples were analyzed for nitrate, soluble reactive phosphate (SRP) and ammonium (NH_4_^+^) using an AutoAnalyzer 3 (Seal Analytical, Inc., Mequon, WI, USA) as described previously (1). Briefly, nitrate was measured using the cadmium reduction technique (2), SRP was measured using the antimonyl tartrate technique (3), and ammonium was measured using the phenol hypochlorite technique (4). Nitrate was calculated as the difference between nitrate+nitrite (NO_x_^−^) and nitrite (NO_2_^−^), which were measured with cadmium reduction and without cadmium reduction techniques, respectively. Samples from remaining time points were analyzed using Hach kits for nitrate (Nitrate TNTplus 835), ammonia (Ammonia TNTplus 830) and SRP (Phosphorus TNTplus 843) (Hach, Loveland, CO, USA) following the manufacturer’s instructions.

References

1. A. McCormick, T. J. Hoellein, S. A. Mason, J. Schluep, and J. J. Kelly, Environ Sci Technol 48:11863–11871, 2014, <https://doi.org/10.1021/es503610r>).
2. APHA, Standard Methods for the Examination of Water and Wastewater, 20th ed., 1998.
3. J. Murphy and J. P. Riley, Anal Chim Acta 27:31–36, 1962.
4. L. Solarzano, Limnol Oceanogr 14:799–801, 1969.
