# Supplementary material for: Taxon-Driven Functional Shifts Associated with Storm Flow in an Urban Stream Microbial Community
Source: mSphere. 2018 Jul 5;3(4):e00194-18. doi: 10.1128/mSphere.00194-18 (PMC6034075; doi:10.1128/mSphere.00194-18)
Supplement: TABLE S2 [file sph004182588st2.docx]

| 16S rRNA gene library^a^ | No of sequences after quality filtering (forward read only) | Median sequence length (bp) | Goods coverage estimate^b^ | OTU richness (# OTUs)^b^ |
| --- | --- | --- | --- | --- |
| AfterRain.Oct2013.A | 141,420 | 241 | 0.77 | 5,297 |
| AfterRain.Oct2013.B1 | 19,313 | 206 | 0.80 | 5,018 |
| AfterRain.Oct2013.B2 | 59,679 | 249 | 0.79 | 5,011 |
| July2014.A | 64,038 | 247 | 0.85 | 4,025 |
| July2014.B | 73,386 | 252 | 0.85 | 3,899 |
| BeforeRain.Oct2013.A | 34,150 | 289 | 0.87 | 3,695 |
| BeforeRain.Oct2013.B1 | 24,075 | 197 | 0.85 | 3,988 |
| BeforeRain.Oct2013.B2 | 28,635 | 290 | 0.88 | 3,503 |
| Oct2014.A | 38,619 | 289 | 0.89 | 3,211 |
| Oct2014.B | 35,751 | 293 | 0.90 | 3,124 |
| July2015.A | 54,255 | 256 | 0.89 | 3,277 |
| July2015.B | 66,487 | 253 | 0.88 | 3,428 |
| Effluent.Oct2013 | 33,272 | 292 | 0.85 | 4,100 |

^a^ Letters ‘A’ and ‘B’ following a sample ID represent biological replicate libraries for a sampled time point, and numbers ‘1’ and ‘2’ following these letters represent sequencing replicates

^b^ Goods coverage estimate and OTU richness were calculated after subsampling each library to the smallest library size
