# Supplementary material for: Taxon-Driven Functional Shifts Associated with Storm Flow in an Urban Stream Microbial Community
Source: mSphere. 2018 Jul 5;3(4):e00194-18. doi: 10.1128/mSphere.00194-18 (PMC6034075; doi:10.1128/mSphere.00194-18)
Supplement: TABLE S3 [file sph004182588st3.docx]

**Table S3: Sequencing statistics for the metagenomes used in the study**

| Metagenome Library | No of paired-end reads after quality filtering (million) | Read  Length (bp) | Estimated community coverage^a^ | No of contigs (contig size > 500 bp) | Contig N50 (bp) | Assembly efficiency  (for contigs > 500 bp)^b^ |
| --- | --- | --- | --- | --- | --- | --- |
| Before Rain1 | 8.74 | 150 | 52% | 98708 | 1283 | 29-59% |
| Before Rain2 | 16.21 | 100 | 61% | 68202 | 1403 | 23-45% |
| After Rain1 | 9.26 | 150 | 47% | 97165 | 1224 | 24-49% |
| After Rain2 | 4.06 | 100 | 36% | 5141 | 1068 | 6-13% |

^a^ Community coverage estimates were obtained from quality filtered reads using Nonpareil^c^

^b^ Assembly efficiency is an estimate of the extent a metagenomic library is represented by its assembled contigs (size >500 bp). This was calculated using the following formula:

Assembly efficiency = $\left[ \left\{ \sum_{i} ({contig}_{i} length\times{contig}_{i} coverage) \right\}\div metagenome size \right]\times100$

Where contig_i_ coverage is the average number of reads that map to contig_i_ from the corresponding metagenome (sum of lengths of reads that map to contig_i_/contig_i_ length) and metagenome size is the total number of base pairs from the library used in the assembly. Given the sequencing insert length for the paired end reads (600 bp for 2x150bp libraries and 500 bp for 2x100bp libraries) for these assembled libraries and the variable length of the contigs (> 500 bp), we estimate that some contigs would represent both the paired-end reads while some would only represent one, so the actual number of bases used in the assembly process should be somewhere between the following numbers –

(total number of basepairs in the paired reads file/2) - (total number of basepairs in the paired end reads file). This leads to the estimated range for our calculated assembly efficiency.

^c^L.M. Rodriguez-R and K. T. Konstantinidis, Bioinformatics 30:629–635, 2014, https://doi.org/10.1093/bioinformatics/btt584
