# Supplementary material for: Taxon-Driven Functional Shifts Associated with Storm Flow in an Urban Stream Microbial Community
Source: mSphere. 2018 Jul 5;3(4):e00194-18. doi: 10.1128/mSphere.00194-18 (PMC6034075; doi:10.1128/mSphere.00194-18)
Supplement: TABLE S4 [file sph004182588st4.docx]

**Table S4: Rare species in before rain microbiome that were in the abundant fraction after rain**

| Species | Relative abundance in after rain microbiome^a^ (%) |
| --- | --- |
| *Francisella tularensis* | 0.78 |
| *Candidatus Nitrospira defluvii* | 0.41 |
| *Simkania negevensis* | 0.32 |
| *Legionella longbeachae* | 0.29 |
| *Legionella drancourtii* | 0.25 |
| *Parachlamydia acanthamoebae* | 0.23 |
| *Chlamydia psittaci* | 0.16 |
| *Micavibrio aeruginosavorus* | 0.15 |
| *Chlamydia trachomatis* | 0.14 |
| *Arcobacter sp. L* | 0.14 |
| *Fluoribacter dumoffii* | 0.13 |
| *Neisseria meningitidis* | 0.11 |
| *Rickettsia endosymbiont of Ixodes scapularis* | 0.1 |
| *Enterococcus faecalis* | 0.1 |

^a^ Abundances for each taxa are relative to the total number of sequences characterized by MyTaxa in the after rain library
